# Allopathy

**Published:** 1882-01

**Authors:** 


### Editorial.

### "ALLOPATHY."

It is a matter of surprise and regret that many well-informed physicians should habitually employ this term of reproach to designate a class of medical practitioners who do not recognize or practice any of the exclusive systems of medicine now so much in vogue.

It is derogatory to the dignity of a scientific physician, and offensive to his sense of propriety and justice, to have such a descriptive epithet applied to his calling, and it argues either disgraceful ignorance or inexcusable carelessness upon the part of any member of the regular profession who makes use of the expression.

Medical men may properly be divided into two grand classes—physicians, and practitioners of exclusive systems. To the latter class belong the disciples of Hahnemann, eclectics, reformed, hydropaths, Thompsonians, root, urine and steam doctors, besides a long list of the unclassified; called into existence by the necessities of an unrelenting poverty, or the promptings of a greedy avarice, and sustained by the prejudices of the ignorant or the credulity of the superstitious.

The educated and scientific physician, of course, accepts no cardinal dogma; acknowledges no narrow creed; recognizes no arbitrary limit; belongs to no sect and tolerates no restriction. He employs any and all of the means that his experience or his reason teaches him to be useful in combatting disease. He refuses to avail himself of nothing that offers relief or promises benefit. He exacts of the earth, the air and the seas agencies which he commands, and, while he holds fast to that which is good, he is ever reaching out for other and better means of cure. Hence the gross impropriety—the utter absurdity of efforts to group such men into a "school," or to define their belief or describe their practice by a word! They are physicians; nothing more and nothing less. In its widest sense that term covers the ground; no other can.

So, while we have homœopaths who yield abject obedience to fixed formulas, and pursue their narrow round; and eclectics, who profess abhorrence of "minerals"; besides the reformed and a host of the deformed, we may well say, presenting every variety of mental and moral obliquity, we happily have no allopathists. Let the term become obsolete; it is a misnomer. Let it die of sheer neglect; it is an offense. It serves only to suggest an error and to perpetuate a delusion.

The people, including the intelligent and cultivated classes, are grievously misinformed respecting this whole subject, and we hold that it is the duty of the physician—doctor means teacher—to enlighten them, in season and out of season, to teach them, by proper means and in a worthy manner, that the physician belongs to no "school" and practices no "pathy." It is the province of the physician to inform them that all practitioners of exclusive systems place themselves outside of the pale of professional recognition and of professional confidence by erecting around themselves arbitrary boundaries, and by declaring their adherence to absurd tenets. They claim, at least, to be thus hedged about, and call it conservatism. If they are true to the faith which they profess, the limits to their usefulness are too circumscribed to be trustworthy. If they are not sincere in their professions, are they then the more entitled to respect and esteem?

